# Harnessing Social Media for Substance Use Research and Treatment

**DOI:** 10.4172/2329-6488.1000238

**Published:** 2016-05-02

**Authors:** Benjamin S Crosier, Lisa A Marsch

**Affiliations:** Center for Technology and Behavioral Health, Dartmouth College, USA

## Short Communication

It is not news that drug addiction is still one of the largest problems we face as a nation, despite decades of attempted social, legal, and medical solutions. New synthetic drugs of abuse seem to be emerging regularly, with substances like Flakka sending unsuspecting users into terrifying and dangerous experiences. Prescription and street opioids are ravaging unsuspecting communities, importing big city problems to bucolic rural locales. Nearly one in 10 (8.4%) American adults confronted a substance use disorder in the past year [1], resulting in an array of personal hardships ranging from the disruption of relationships to death. If that fact alone provides insufficient motivation to engender change, consider the fact that tobacco, alcohol, and illicit drug use cost the United States U$700 billion annually [2], which is $244 billion more than we spend on Medicaid. And, this problem is not unique to the U.S but is a global problem. The United Nations Office of Drugs and Crime estimates that there are between 16 million and 39 million problem substance users across the world, with only one in six having access to, or receiving, treatment [3]. Drastically new approaches for research and treatment are clearly required to address this persistent and evolving worldwide need.

Social media can serve as a foundation for some of these novel technology-based methods. Social media, such as Facebook and Twitter, are not temporary trends or mere gossip machines – they constitute highly scalable and efficient tools for reaching those experiencing problem drug use. Although the big players of the day in social media services are tech juggernauts, they are susceptible to failure, as was seen with MySpace. However, social media itself, regardless of the rise and fall of particular services, will remain a global phenomenon for decades to come [4]. Social media also provides an invaluable source of data in and of itself. Academic behavioral health researchers, including those in the realm of substance abuse, have only barely tapped their potential.

Almost everybody uses the Internet in America. Eight-four percent of adults are online [5]. The fact that a staggering 96% of the 18-29 year old demographic are online suggests a near perfect eventual saturation. This translates to social media, with 74% of these online adults using one or more social media services, a 66% increase in the last decade [5]. Users come from every single demographic – and not just rich, white, educated suburbanites. Facebook has 1.59 billion users worldwide [6], which translates to over 1.5% of humans that have ever lived on this planet, thereby providing a service with a larger population than any country on the globe. This statistic is especially impressive in light of the fact that the most populous country (China) currently bans the use of Facebook Although Facebook is the most popular social media platform, each service offers a different experience, promoting a unique demographic complexion for each service’s user base. For example, younger people are leaving Facebook for Snapchat and Instagram, while Baby Boomers are the fastest growing demographic on Facebook [5]. “Black Twitter” highlights the highly active and tight-knit community of African American users on Twitter, as well as the power of the numerous grassroots political movements it spawned, such as #BlackLivesMatter [7].

The legion of users of each service can be engaged for research and treatment. This can be done either organically (e.g., posting to public groups, digital word of mouth) or through advertising. Surveys can be distributed to a vast population for a low cost. The raw data generated by users can be used either alone or in conjunction with other data sources (e.g., surveys) to address endless scientific questions. Those in need of help for problematic substance use can be recruited to in-person and online treatment solutions. Further, online interventions can be augmented with therapeutically focused social support by leveraging the inherently ubiquitous and interactive nature of social media. Such next-generation empirically validated treatment solutions could transform the current system of care delivery.

Current exciting work highlights the promise of tapping social media for treatment and research. The use of social media advertising as a participant recruitment tool in substance use research is just now starting to gain some traction, much of it inspired by Danielle Ramo’s 2012 study that collected substance use data from young adults [8]. The authors of the study distributed a web-based survey to tens of millions of potential participants, obtaining consent from N=5,237, with each participant costing only $4.28. Although some other studies have used Internet advertisements, this study was the first that used Facebook advertisements to their truest potential within the realm of drug abuse research. This methodological paragon inspired some of our own work – the author BC was able to collect data on nearly 3,000 marijuana users in just a few days for $0.27 per person [9]. The promise of technology is also evident in treatment. The author LM served as one of the lead developers of Square2 [www.square2.co], a mobile behavior change tool that employs the fundamental principles of the science of behavior change, including the power of social media-based support for behavior change for a way array of behaviors, into a single mobile platform. The power of the social supports is underscored by the countless support groups that have sprung up on the web and nearly every social media service that represent nearly every type of substance use problems. Beyond research recruitment and intervention, social media data itself can be used for research purposes – such as using text and geospatial information from Twitter posts to track disease activity and sentiment related to the H1N1 epidemic [10,11], or predicting the substance use behavior of Facebook members from what they have “Liked” [12].

Given the enormous opportunity to leverage social media for unprecedented insight and resource delivery, [13] a large amount of attention and resources should be focused on future research in this area. Scientists conducting survey research should consider using social media advertisements as a collection tool as part of their research agenda in light of the low cost, ease of use, and advanced targeting options that can be used to augment, or create entirely new, sampling strategies. Future web-based interventions should take advantage of these existing platforms. Social media professionals have already completed the necessary interface and experience design work to create platforms with massive acceptance and utilization. These platforms offer an unparalleled avenue for engagement. However, the most striking opportunity for potential progress is arguably the use of fine-grained social media data itself. The text, images, video, and interactions (e.g., commenting, messaging, liking and sharing) that are produced in the context of social media serve as automatically logged records of our thoughts, emotions, and behaviors. This can all be used to learn a great deal about substance use, ranging from opportunities for population-wide epidemiological surveillance to highly accurate predictive personal analytics. Much as a market researcher at Target was famously able to predict customer pregnancy using purchase patterns with amazing accuracy, researchers may one day be able to spot behavioral health problems in at-risk individuals on the horizon before they happen.

These endeavors will become even more powerful when social media data are combined with the Internet of Things [14]. Perhaps someday incredibly accurate predictive analytics that are powered be a confluence of insights from multiple data streams could be integrated into automated treatment systems that are highly tailored to each individual. This may provide an entirely new way to engage the large proportion of individuals with problematic substance use who do not engage in our traditional systems of substance abuse treatment. These systems could automatically help build support, sculpt healthy social networks, promote healthy behaviors, promote mental health, and provide access to information and resources. Multiple “robo advisers” exist to manage retirement investments. Investors select an investment risk level with which they are comfortable, and an algorithm takes care of the rest. Similar systems could be built for behavioral health, with mobile communication systems that rely on social media serving as a tool to guide better behavioral health decisions. Although, this speculation may seem beyond the realm of possibility to some, given the current trends in digital technologies and health, this is likely to become part of our reality in the next decade. But, surely the most astonishing advances in social media-powered mobile health as applied to substance research remain out the realm of current imagination.

Substance misuse is a significant problem at personal, national, and global levels, and barriers to research and treatment impede progress in addressing this preventable public health concern. We do not mean to offer the glib suggestion that social media is the panacea for addiction. It is not. However, in order to deal with emerging problems like the national opioid epidemic, we need to harness agile, scalable, and highly efficient approaches to consumer engagement, research, and treatment. Doing so requires expanding technological access by promoting pervasive broadband availability and by providing assistance to low income families, as was done with past privately funded efforts for telephone access. Additional limitations of using technology-based behavioral health research methods and interventions are found in populations that may possess limited technological competence. These include elderly populations, although this cohort effect will eventually diminish, and those with cognitive impairments. However, technology presents potential solutions for these populations too. It is our belief that a family of next generation of approaches will center on mobile health technologies based in social media. In 50 years, we will be able to look back to current research and treatment approaches and recognize the advances that have been achieved with leveraging digital technologies in entirely new and disruptive ways. Technology-based interventions, especially those that harness the social power and invaluable data drove of social media and related digital communication platforms, could represent the most substantial leap in behavioral health care since the dawn of modern psychopharmacology in the 1950s.
